# Treatment-limiting decisions in patients with severe traumatic brain injury in the Netherlands

**DOI:** 10.1016/j.bas.2024.102746

**Published:** 2024-01-03

**Authors:** Inge A.M. van Erp, T.A. van Essen, Erwin J.O. Kompanje, Mathieu van der Jagt, Wouter A. Moojen, Wilco C. Peul, Jeroen T.J.M. van Dijck

**Affiliations:** a University Neurosurgical Center Holland, Leiden University Medical Center, Haaglanden Medical Center and HaGa Hospital, Leiden and The Hague, the Netherlands; b Division of Neurosurgery, Department of Clinical Neurosciences, University of Cambridge and Addenbrooke's Hospital, Cambridge, United Kingdom; c Department of Intensive Care Adults, Erasmus MC – University Medical Center, Rotterdam, the Netherlands; d Department of Ethics and Philosophy of Medicine, Erasmus MC – University Medical Center, Rotterdam, the Netherlands

**Keywords:** Treatment-limiting decision, Severe traumatic brain injury, Prognosis

## Abstract

**Introduction:**

Treatment-limiting decisions (TLDs) can be inevitable severe traumatic brain injury (s-TBI) patients, but data on their use remain scarce.

**Research question:**

To investigate the prevalence, timing and considerations of TLDs in s-TBI patients.

**Material and methods:**

s-TBI patients between 2008 and 2017 were analysed retrospecively. Patient data, timing, location, involvement of proxies, and reasons for TLDs were collected. Baseline characteristics and in-hospital outcomes were compared between s-TBI patients with and without TLDs.

**Results:**

TLDs were reported in 117 of 270 s-TBI patients (43.3%) and 95.9% of deaths after s-TBI were preceded by a TLD. The majority of TLDs (68.4%) were categorized as withdrawal of therapy, of which withdrawal of organ-support in 64.1%. Neurosurgical intervention was withheld in 29.9%. The median time from admission to TLD was 2 days [IQR, 0–8] and 50.4% of TLDs were made within 3 days of admission. The main reason for a TLD was that the patients were perceived as unsalvageable (66.7%). Nearly all decisions were made multidisciplinary (99.1%) with proxies involvement (75.2%). The predicted mortality (CRASH-score) between patients with and without TLDs were 72.6 vs. 70.6%. The percentage of TLDs in s-TBI patients increased from 20.0% in 2008 to 42.9% in 2012 and 64.3% in 2017.

**Discussion and conclusion:**

TLDs occurred in almost half of s-TBI patients and were instituted more frequently over time. Half of TLDs were made within 3 days of admission in spite of baseline prognosis between groups being similar. Future research should address whether prognostic nihilism contributes to self-fulfilling prophecies.

## Highlights

• Treatment limiting decisions occur in almost half of severe traumatic brain injury patients.
• These decisions are generally made within three days of admission.
• The prevalence of treatment limiting decisions seem to increase over years, independent from prognostic variables.
• Future research should address whether prognostic nihilism contributes to self-fulfilling prophecies.

## 1 Introduction

Traumatic brain injury (TBI) is a heterogeneous disease and its different degrees of injury severity cause a wide variety of global healthcare and socioeconomic problems (Maas et al., 2017; Steyerberg et al., 2019). Patients sustaining a severe TBI (s-TBI), usually defined by an early Glasgow Coma Score (GCS) of 3–8, generally show the highest mortality rates (30%–50%) and substantial long-term consequences in the majority of survivors (Marmarou et al., 2007; Beck et al., 2017; Honeybul et al., 2013; Fountain et al., 2017; Chieregato et al., 2017).

Multidisciplinary teams aim to ease this burden by making acute care decisions and initiating individualized treatment strategies (Carney et al., 2017). It is, however, difficult to find the optimal balance between continuing treatments that will not be effective and prematurely limiting treatments that could have resulted in an acceptable outcome (Honeybul et al., 2013; van et al., 2019a). Scarce high-quality evidence on treatment effectiveness, major uncertainties in prognostication, and disease heterogeneity are the likely causes of decision-making difficulties in s-TBI and result in treatment variation and non-conformance to guidelines (van et al., 2019a; Han et al., 2014; Turgeon et al., 2013; van Essen et al., 2022). Although poorly studied, variation in treatment decisions and treatment limitations are likely explained by a physicians’ personal and clinical experience, religious background, cultural values, regional legislation, and valuation of predicted outcome (van et al., 2019a). These factors give rise to unique decision-making contexts and thereby substantial variation in acceptance and occurrence of TLDs around the globe (Mark et al., 2015), in Europe (van Veen et al., 2020, 2021), and even within countries (Turgeon et al., 2011). Especially the Dutch attitude regarding acceptance of TLDs is different from many other countries. Also proxies from certain religions might not always accept initiated or proposed TLDs.

Despite challenges in decision-making, treatment-limiting decisions (TLDs) are frequently used and as much as 63%–86% of deaths after s-TBI occur following TLDs (Leblanc et al., 2018). They are considered to be inevitable and morally justified in certain s-TBI patients, but may also be premature, especially considering the uncertainty in prognostication and significant variation in TLD approaches (van et al., 2019a; Turgeon et al., 2011; Lazaridis, 2019). In addition, TLDs could also bias mortality rates in clinical trials and contribute to incorrect nihilistic conclusions and self-fulfilling prophecies (van Veen et al., 2021; Mertens et al., 2022).

Studying TLDs is crucial to understand outcome after s-TBI, but comprehensive overviews of clinical practice are scarce in literature. Therefore, this study aims to provide insight in the prevalence, timing, and reasons for TLDs in patients with s-TBI in a Dutch trauma care setting.

## 2 Material and methods

This study was conducted and reported following the criteria of the Strengthening the Reporting of Observational Based Studies (STROBE) statement (von Elm et al., 2007). Use of anonymized retrospective data according to national legislation was approved by the responsible ethics review board.

### 2.1 Study design, setting and patients

This retrospective cohort study was conducted at two level one trauma centers. Patients were identified by screening the electronic hospital registration systems using TBI specific diagnostic codes. All consecutive s-TBI patients with GCS 3–8 on admission were included between 2008 and 2017. The lowest GCS score documented at the Emergency Room (ER) or the last reliable score before intubation and/or sedation was used to determine eligibility. Patients were excluded when no acute cerebral traumatic abnormalities were diagnosed on computed tomography (CT) scan or when essential data were not available in the medical records (e.g. GCS on admission).

### 2.2 Clinical data

Data were independently collected and stored in a predefined database. Collected variables included demographics, patient and trauma specific information and in-hospital parameters. Comorbidities present before sustaining s-TBI were defined by the ASA classification: (I) a normal healthy patient; (II) a patient with mild systemic disease; (III) a patient with severe systemic disease; (IV) a patient with severe systemic disease that is a constant threat to life (Schupper et al., 2020). Characteristics of the first CT-scan on admission were collected. A large epidural hematoma was defined with a thickness greater than 15 mm (Bullock et al., 2006a). A large acute subdural hematoma is defined with a thickness greater than 10 mm (Bullock et al., 2006b). The Corticosteroid Randomisation after Significant Head Injury (CRASH) prognostic model was used to calculate the baseline probability of mortality and unfavourable outcome at 6 months. The in-hospital parameters included the medical and surgical interventions performed, mortality, Intensive Care Unit (ICU) and hospital length of stay (LOS) and discharge location of the patients. Given the aim of this study, no post-discharge follow-up was performed.

### 2.3 Treatment limiting decisions

TLDs were categorized into different categories for analyses. First, as generally used in literature, TLDs were divided in (1) withholding life-saving interventions, and (2) withdrawing life-sustaining measures. Because it is possible to limit more than one treatment per patient, several types were distinguished: (1) withholding ICU admission, (2) withholding neurosurgical intervention, (3) withholding escalation of ICP targeted treatment, (4) do not resuscitate order, (5) withholding of organ-support, (6) withdrawal of organ-support, (7) withdrawal of intracranial pressure (ICP) targeted treatment and (8) withdrawal of nutrition support. Patient location (ER, operating theatre (OR), ward, or ICU) and neurological state (GCS and pupillary abnormalities) were retrieved at time of TLD. The time from hospitalization to TLD and from TLD to death was collected in days.

The main reason for TLDs was collected from electronic patient files. Reasons were coded based on actual text, or, when unclear, a senior authors' interpretation (JD) of the text, and categorized as follows: (1) Neurologic: unsalvageable; (2) Neurologic: expected very bad quality of life (QoL) (physician initiative); (3) Neurologic: expected very bad QoL (proxy initiative); (4) Patients' (reconstructed) preference; (5) Proxies’ preference or (6) Other (reason should be specified). Proxies could state their preference, but the final decision to initiate TLDs was always taken by the responsible physician of treatment team considering the best interests of the patient. Based on chart review, key aspects of the decision-making processes were registered, including who made the decision: (1) multi-disciplinary or (2) by a single physician. Moreover, the involvement of proxies was reported. Documentation indicated whether or not there were disagreements or conflicts between the treatment team and proxies, including involvement of clinical ethics committee or palliative care team consultation.

### 2.4 Statistical analysis

Categorical data were presented as absolute numbers and percentages, whereas continuous variables were reported as medians and interquartile ranges (IQR) [25–75]. Baseline characteristics, CRASH baseline prognosis and in-hospital outcomes were described for patients with or without TLD. Chi-square test or Fisher's exact test were used for categorical variables and unpaired *t*-test or Wilcoxon rank sum test for continuous data as appropriate. ANOVA test was used to test differences in the CRASH baseline prognosis score over years. A p-value of <0.05 was considered statistically significant. All analyses were performed using IBM's Statistical Package for Social Sciences (SPSS) version 25.

## 3 Results

**Fig. 1:**
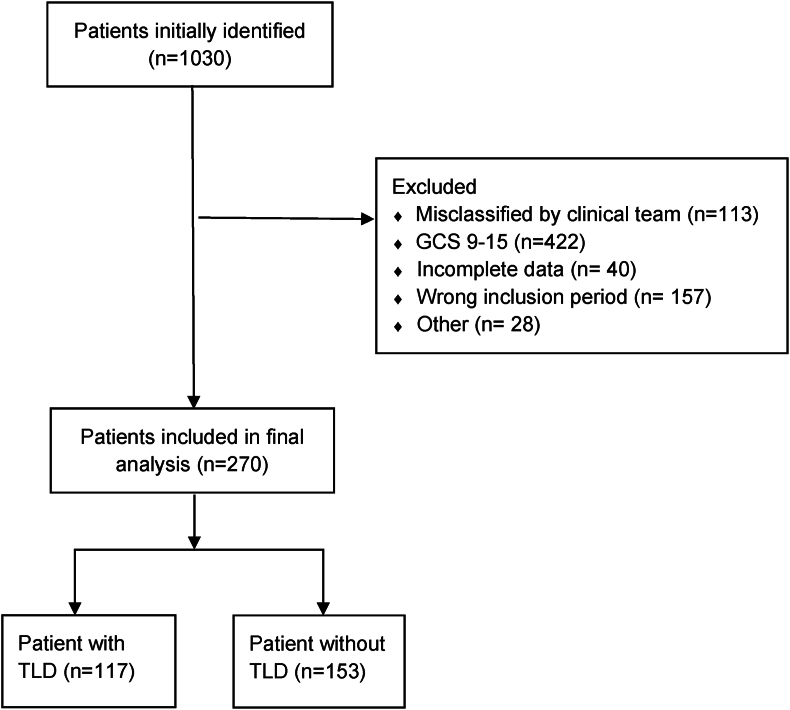
Patient selection flowchart AbbreviationsTBI: traumatic brain injury, GCS: Glasgow Coma Scale, TLD: treatment limiting decision.

**Fig. 2:**
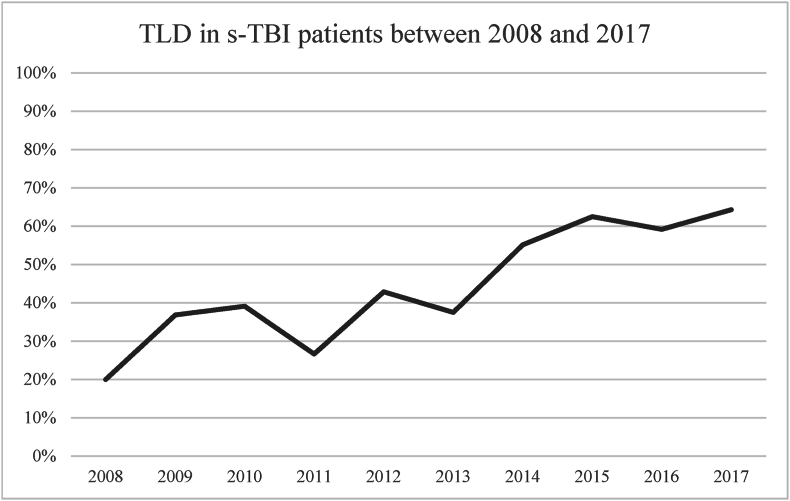
Percentage of TLDs in s-TBI patients between 2008 and 2017 Abbreviationss-TBI: severe traumatic brain injury, TLDs: treatment limiting decisions.

**Fig. 3:**
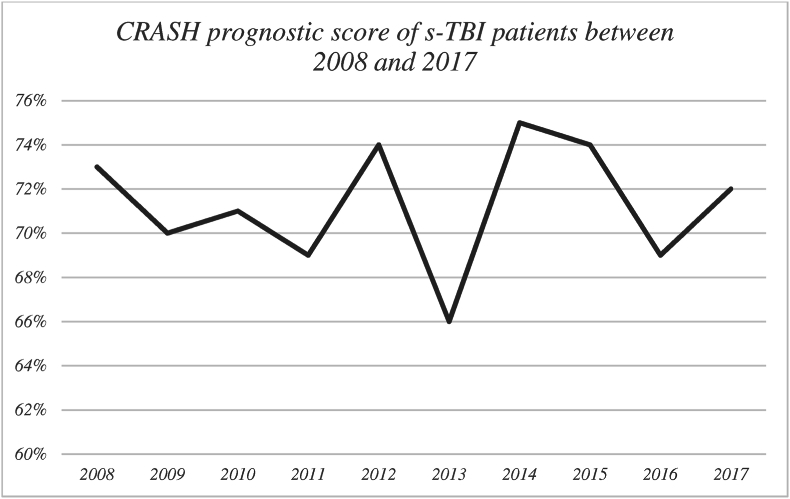
CRASH baseline prognostic score between 2008 and 2017 Abbreviations: s-TBI: severe traumatic brain injury ANOVA test p-value = 0.21.

A total of 270 s-TBI patients were included from the 1030 patients that had a diagnostic TBI code (Fig. 1). TLDs were made in 117 of the 270 (43.3%) included s-TBI patients and 95.9% of deaths after s-TBI followed a TLD. The percentage of TLDs in s-TBI patients increased from 20.0% in 2008 to 42.9% in 2012 and 64.3% in 2017 (Fig. 2). The CRASH prognosis risk score of unfavourable outcome at 6 months did not significantly differ over the years (73.1% in 2008 to 73.9 in 2012 and 72.5 in 2017; p 0.31; Fig. 3).

All patients with TLD, except one, died in the hospital. One patient was transferred to a hospice after withholding early neurosurgical treatment. In the majority of patients (68.4%), treatment was initiated, but withdrawn later. In the remaining 31.6% of patients, treatment was withheld at presentation. Regarding the types of TLD, withdrawal of organ-support (64.1%) and withholding of neurosurgical intervention (29.9%) were the two most frequent decisions (Table 1).

**Table 1:** Types of treatment-limiting decisions in all the TLD-group.

Type of TLD	TLD (n = 117)∗
Withholding, %	
Access to ICU	2 (1.7)
Neurosurgical intervention	35 (29.9)
No escalation of ICP targeted treatment	7 (6.0)
DNR order	23 (19.7)
Organ-support (ventilator)	0 (0.0)
Withdrawal, %	
Organ-support (ventilator)	75 (64.1)
ICP targeted treatment	3 (2.6)
Nutrition support	2 (1.7)

**Abbreviations**: TLD: treatment-limiting decision, ICP: intracranial pressure, ICU: Intensive Care Unit, DNR: Do Not Resuscitate.

∗ Several TLDs can be addressed to one patient.

Most of the TLDs were made at the ICU (78.6%), whereas 12.8% of TLDs were made at the ward, 8.5% in the ER and in 1 patient the TLD was made in the OR **(**Table 2**)**. At time of TLD, the median GCS was 3 [IQR, 3–4] and 82.9% of patients showed pupillary abnormalities. The median time from admission to TLD was 2 days [IQR, 0–8]. Over fifty percent of TLDs were made within the first three days after admission and 28.2% of these TLDs were even made within the first day of hospital admission. The median time from TLD to death was 1 day [IQR, 0–1], although intervals from TLD to death of 10, 17 and 26 days have also been reported.

**Table 2:** Details characteristics of TLDs.

	TLD (n = 117)
Location of TLD, %	
ER	10 (8.5)
ICU	92 (78.6)
Ward	15 (12.8)
OR	1 (0.9)
GCS at time of TLD, median [IQR]	3 [3-4]
Pupillary abnormality at time of TLD, %	97 (82.9)
Time from admission to TLD (days), median [IQR]	2 [0–8]
TLD within 72 h after injury, %	59 (50.4)
TLD in first day, %	33 (28.2)
Time from TLD to death (days), median [IQR]	0 [0–1]
Reason for TLD, %	
Neurologic: not possible to survive	78 (66.7)
Neurologic: very bad QOL (proxy initiative)	17 (14.5)
Neurologic: very bad QOL (physician initiative)	19 (16.2)
Patients' (reconstructed) preference	1 (0.9)
Proxies' preference	0 (0.0)
Other	2 (1.7)
Decision for TLD by, %	
Multidisciplinary	116 (99.1)
Single physician	1 (0.9)
Relative involvement	88 (75.2)

**Abbreviations**: TLD: treatment-limiting decision, ER: Emergency Room, ICU: Intensive Care Unit, OR: Operating Room, GCS: Glasgow Coma Scale, QOL: Quality of life.

The main reason for a TLD (66.7%) was that the patient was considered unsalvageable. Other important reasons were an unacceptable QoL as presumed by the physician (16.2%) or the proxy (14.5%). The two patients that were categorized as ‘other’ were patients for whom a TLD was made based on non-survivable extra-cranial injuries. Almost all TLDs were made multidisciplinary (99.1%) with a large percentage of proxy involvement (75.2%).

Patients with TLD (N = 117) were older compared to patients without TLD (N = 153) (58 years vs. 45; p < 0.001), but the groups did not differ with regard to gender (female: 32.5 vs. 37.9%; p = 0.386) **(**Table 3**).** Patients in the TLD group had a lower median GCS (3 [IQR, 3–5] vs. 4 [3-7]; p = 0.014), more pupillary abnormalities (bilateral 53.0 vs. 21.7%; p < 0.001), and more pre-injury comorbidities (ASA classification I: 47.9 vs. 56.2%; II: 18.8 vs. 24.2%; III: 23.1 vs. 16.3%; IV: 10.3 vs. 3.3%; p = 0.002) at presentation. The TLD-group presented with more extensive subdural hematomas (large (>10 mm): 68.4 vs. 34.0%; p < 0.001), subarachnoid hemorrhage (basal and cortical: 28.2 vs. 7.2%; p < 0.001), brainstem lesions (18.8 vs. 2.6%; p < 0.001), herniation (34.2 vs. 15.0%; p < 0.001), contusions (23.9 vs. 13.7%; p = 0.036), depressed skull fractures (22.2 vs. 8.5%; p = 0.003), compressions of the basal cisterns (53.0 vs. 34.6%; p = 0.009) and presence of midline shift (66.7 vs. 46.4%; p = 0.007) on the first CT-scan. The CRASH prognosis risk score of 14-day mortality and unfavourable outcome at 6 months was 72.6% in the TLD patients and 70.6% in the non-TLD patients (p = 0.71).

**Table 3:** Patient and injury characteristics of TLD and no TLD group.

	TLD (n = 117)	No TLD (n = 153)	P-value
Age, mean (SD)	58 (20.6)	45 (22.4)	<0.001
Sex, %			0.36
Female	38 (32.5)	58 (37.9)	
Male	79 (67.5)	95 (62.1)	
Mechanism of injury, %			0.39
High velocity trauma	44 (37.6)	63 (41.2)	
Direct impact: head against object	12 (10.3)	10 (6.5)	
Ground level fall	28 (23.9)	33 (21.6)	
Fall from height∗	32 (27.4)	42 (27.5)	
Other	1 (0.9)	5 (3.3)	
Extracranial injury∗∗, %			0.27
No	47 (40.2)	44 (28.8)	
Mild	24 (20.5)	41 (26.8)	
Moderate	26 (22.2)	42 (27.5)	
Severe	20 (17.1)	26 (20.0)	
Pre-injury ASA classification, %			0.002
I	56 (47.9)	86 (56.2)	
II	22 (18.8)	37 (24.2)	
III	27 (23.1)	25 (16.3)	
IV	12 (10.3)	5 (3.3)	
ER SBP, mean (SD)	144 (44)	142 (32)	0.69
ER DBP, mean (SD)	81 (29)	80 (23)	0.59
ER HR, mean (SD)	92 (27)	86 (22)	0.06
ER GCS, median [IQR]	3 [3-5]	4 [3-7]	0.01
3-5	90 (76.9)	95 (62.1)	
6-8	27 (23.1)	48 (37.9)	
ER pupillary reflex, %			<0.001
Both reacting	26 (22.2)	74 (48.7)	
One reacting	13 (11.1)	23 (15.2)	
Both unreacting	62 (53.0)	33 (21.7)	
CT characteristics, %			
Epidural hematoma			0.04
*Small*	13 (11.1)	22 (14.4)	
Large***	6 (5.1)	21 (13.7)	
Subdural hematoma			<0.001
*Small*	24 (20.5)	51 (33.3)	
Large***	80 (68.4)	52 (34.0)	
Subarachnoid hemorrhage			<0.001
*Basal*	45 (38.5)	63 (41.2)	
*Cortical and basal*	33 (28.2)	11 (7.2)	
DAI	4 (3.4)	17 (11.1)	0.04
Brainstem lesion	22 (18.8)	4 (2.6)	<0.001
Herniation	40 (34.2)	23 (15.0)	<0.001
Contusion			0.04
*Small*	26 (22.2)	54 (35.3)	
*Large*	28 (23.9)	21 (13.7)	
Depressed skull fracture	26 (22.2)	13 (8.5)	0.003
Compression basal cistern	62 (53.0)	53 (34.6)	0.009
Presence of midline shift	78 (66.7)	71 (46.4)	0.007
CRASH prognosis mortality 100%	85 (72.6)	108 (70.6)	0.71
CRASH prognosis unfavourable outcome 100%	85 (72.6)	108 (70.6)	0.71

**Abbreviations**: TLD: treatment-limiting decision, SD: standard deviation, ER: Emergency Room, SBP: systolic blood pressure, DBP: diastolic blood pressure, HR: heart rate, GCS: Glasgow Coma Scale, CT: Computed tomography, DAI: diffuse axonal injury.

∗ >1 m/5 stairs.

∗∗ Mild extracranial injury: home without treatment, moderate: admission to ward, severe: admission to ICU.

∗∗∗ Large epidural hematoma >15 mm, large subdural hematoma >10 mm.

The median ICU LOS and median hospital LOS were longer in the non-TLD-group **(**6 days [2-14] vs. 3 [1-8]; p<0.001 and 20 [9-41] vs. 4 [2-10]; p < 0.001 respectively, Table 4). These patients also had more neurologic (23.5% vs. 12.8%; p < 0.001) and systemic (54.2% vs. 18.8%; p < 0.001) complications. There were no significant differences in the total number of neurosurgical interventions between the TLD and non-TLD group.

**Table 4:** Interventions and hospital outcomes of TLD and no TLD-group.

	TLD (n = 117)	No TLD (n = 153)	P-value
Neurosurgery, %			
ICP monitor	55 (47.0)	77 (50.3)	0.589
EVD	11 (9.4)	18 (11.8)	0.534
Craniotomy	31 (26.5)	54 (35.3)	0.123
Decompressive craniectomy, %			0.120
Unilateral	31 (26.5)	25 (16.3)	
Bilateral	1 (0.9)	1 (0.7)	
Complications, %			
Neurologic	15 (12.8)	36 (23.5)	0.026
Systemic	22 (18.8)	83 (54.2)	<0.001
ICU length of stay, median [IQR]	3 [1-8]	6 [2-14]	<0.001
Hospital length of stay, median [IQR]	4 [2-10]	20 [9-41]	<0.001
In-hospital mortality, %	116 (99.1)	6 (3.9)	<0.001
Reason of death, %			<0.001
Initial intracranial injury	107 (91.5)	4 (57.1)	
Extracranial injury	2 (1.7)	1 (14.3)	
Medical complications	7 (6.0)	1 (14.3)	
GCS at discharge, median [IQR]	3	9 [6-13]	
Discharge destination, %			
Home	–	36 (23.5)	
Rehabilitation	–	76 (49.7)	
Nursing home	1 (0.9)	12 (7.8)	
Other hospital	–	23 (15.0)	

**Abbreviations**: TLD: treatment-limiting decision, ICP: intracranial pressure, EVD: external ventricular drain, ICU: Intensive Care Unit, GCS: Glasgow Coma Scale.

A total of 122 of the 270 s-TBI patients died during hospitalization and in 116 patients (95.9%) of these patients a TLD was made. The main reason of death in both groups was the initial intracranial injury (TLD: 91.5% and non-TLD: 57.1%), whereas a relatively small proportion of patients died due to extracranial injuries (TLD: 1.8% and non-TLD: 14.3%), or medical complications (TLD: 6.0% and non-TLD: 14.3%). Of the patients that received a TLD, 99.2% died in the hospital and 1 patient was transferred to a nursing home and died. Half of the total cohort that survived hospitalization were discharged to a rehabilitation center, whereas 23.5% were discharged to home, 7.8% to a nursing home and the remaining 15.0% were transferred to another hospital. Median GCS at discharge in the non-TLD group was 9 [6-13].

There were two documented cases where the treatment team recommended withdrawal of treatment but proxies refused cooperation. Withdrawal was eventually accepted in one case, but in the other case treatment was withdrawn based on the medical decision of futility of further treatment without final proxy consent. Both refusals were motivated by cultural and religious reasons. There were also three patients where proxies requested to withdraw and/or withhold treatment but the treatment team initially refused. Treatment was withdrawn shortly after in two patients and after a week in the third patient.

## 4 Discussion

Overall, TLDs were made in 43.3% of all s-TBI patients and 95.9% of deaths were preceded by a TLD. Most of the TLDs were made on the ICU (78.6%). The median time from admission to TLD was 2 days, with 50.4% made within the first three days. Moreover, the percentage of TLDs in s-TBI patients increased over time whilst the CRASH prognostic score did not change.

The percentage of TLDs in this cohort was relatively high compared to previous studies. When comparing to general ICU practices, TLDs are reported in 10% and 13% of all ICU admissions (Verkade et al., 2012; Sprung et al., 2003). The higher TLD rate in our study can be explained by the fact that s-TBI patients usually have a worse prognosis than the average ICU patient (Steyerberg et al., 2019). When focussing on s-TBI patients, a Canadian study reported TLDs in 22% of s-TBI patients (Turgeon et al., 2011). In Norway, TLDs were reported in 17% of all admitted s-TBI patients (Robertsen et al., 2017). The high rate of TLDs in our study could be explained by the relatively large proportion of very severely injured patients (i.e. low GCS, pupillary abnormalities) where the decision to withhold or withdraw treatment seemed to be unavoidable. This is supported by the finding that the majority of TLDs were considered in unsalvageable patients. The 96% mortality rate after TLDs in this study was comparable to other studies, supporting the unambiguous impact of TLDs (van Veen et al., 2021; Williamson et al., 2020). Another explanation for the high number of TLDs in this study could be the unique interplay of cultural, social, legal and political attitudes towards end-of-life practices in The Netherlands (van Veen et al., 2021; Janssen, 2002; van der Heide et al., 2017). Allowing TLDs within limitations of the law enables individuals to have more control over their own death and reduce unnecessary suffering. Other countries may view this as controversial, since it challenges traditional beliefs and ethical norms surrounding the value of human life and role of healthcare providers (Mark et al., 2015; van Veen et al., 2020, 2021). This emphasizes the unique Dutch attitude towards these decisions.

Of all TLDs, the more frequently used withdrawal of treatment compared to withholding treatment contradicts earlier reports (Attitudes of critical care medicine, 1992; Sprung et al., 1997). This could be explained by a more aggressive (surgical) treatment strategy in including study centers (van Essen et al., 2017, 2019). In addition, differences in TLDs between physicians, centers, and clinical studies might be further explained by differences in religion, cultural values and clinical training (van Veen et al., 2020; Sprung et al., 2003, 2007).

Most of the TLDs were made in the ICU, comparable to other studies and representing daily clinical practice. In one patient the decision to withdraw treatment was made in the operating room. Due to the acute setting, in many situations is family not present in the first hours after trauma. In this particular patient, family expresses the strong wish of the patient not being operated when located in the OR and therefore surgery was not performed.

The increased percentage of TLDs in s-TBI patients between 2008 and 2017 could be caused by an improvement of TLD registration by treatment teams or a more severe (unmeasured) baseline clinical presentation but could also be a genuine reflection of evolving clinical practice. It may reflect a decreased willingness of clinicians and proxies to accept the prospect of unacceptable outcome for patients (Wilson et al., 2017). The increase of TLDs over the years, independent of baseline prognostic scores, might attest to this possibility. Finding the balance between continuing treatments that may not be effective and prematurely limiting treatments that could have resulted in acceptable outcome is very difficult, especially when there is high uncertainty on best clinical practice (van et al., 2019a).

Because of the uncertainty in early clinical decision-making regarding prognostication and treatment effectiveness, the NeuroCritical Care Society (NCCS) recommends a 72 h observation period to determine clinical response and delay decisions regarding withdrawal of life-sustaining measures (Souter et al., 2015). Other authors also state that adequate acute treatment should not be withheld in the acute phase as there are many uncertainties and the majority of patients cannot reliably be considered unsalvageable (van et al., 2019b; Geurts et al., 2014). Thus, the high number of early TLDs in this study may indicate unjustified prognostic pessimism in some cases. The use of early TLDs could also be explained by clinical nihilism or the unwillingness of acting against a patients' or proxy's best interest by prolonging suffering or achieving survival with an unacceptable outcome (Kitzinger et al., 2013). Another explanation of high early TLDs percentages could be the use of DNR orders (N = 23), which are also TLDs by definition.

The idea that TLDs are based on a single physicians' understanding of the medical, ethical, cultural and religious issues, is outdated (Manalo, 2013; Mazanec et al., 2003). Most TLDs in this study were made in a multidisciplinary fashion and usually with proxy involvement. This is in accordance with the NCCS recommendation of early, frequent and consistent multidisciplinary communication towards proxies about the patient's condition and prognosis and to include a discussion of prognostic uncertainty if appropriate (Souter et al., 2015). A recent European study also advises multidisciplinary discussion before withdrawing or withholding treatment (van Veen et al., 2020). Proxy involvement is essential to reconstruct a patients' wishes and thereby individualize treatment. Also, this could prevent conflicts because of religious or cultural objections to TLDs (de et al., 2011). The overall low rate of TLDs defined by conflicts between treatment team and proxies, is reassuring and in line with other studies (Schaller et al., 2006). In addition, differences in TLDs between physicians, centers, and clinical studies might be further explained by, amongst others, differences in religion, cultural values and clinical training (van Veen et al., 2020; Sprung et al., 2003, 2007).

This study is the first detailed overview of TLDs in s-TBI patients in The Netherlands. This study has some important limitations. First, due to the retrospective study design all data regarding TLDs is based on chart review and therefore, inevitable complexities of these decisions were not be captured. Second, data collection was from 2008 to 2017, thereby potentially not representing up-to-date clinical practice. Also, data was collected from two Dutch trauma centers. When comparing the Netherlands to other European countries, there could be different perspectives on quality of life when confronted with a lifelong severe disability. This might be driven by different cultural and religious backgrounds and limits the generalizability of our results to other countries. Moreover, no data on radiologic characteristics at timing of TLD was collected. The CRASH prognostic score only represent baseline prognosis and it might be plausible that patients in the TLD group have more secondary clinical deterioration.

Future research should address the possible geographical disparities in TLDs and whether (progressive) nihilism drives these decisions. Moreover, this research should elaborate on the radiologic and clinical features at timing of TLD instead of at hospital admission. Moreover, it should incorporate the effect of frailty on TLDs with the aging TBI population, thereby provide more granularity on these difficult decisions. This data will contribute to a more standardized decision tree on TLDs in these critical patients. As the decision to withdraw treatment is associated with mortality, differences within and between centers, treatment groups and individual cases are likely to induce selection bias in the analysis of (international) comparative studies (e.g. clinical trials) (Leblanc et al., 2018). TLD policies should therefore be explicitly reported in trials with TBI patients to improve the external validity and interpretation of trial results.

## 5 Conclusion

TLDs occurred in almost half of s-TBI patients and most of them were made within 3 days despite guidelines recommending a 72-h observation period. Baseline prognosis between patients with and without TLDs were similar. The prevalence of TLDs increased over years, independent from prognostic variables. TLD should be prospectively investigated and included in clinical trials for better interpretation of studies on therapeutic interventions or prognosis.

## Funding

This work was supported by The 10.13039/501100000780European Union seventh Framework Program (grant 602,150) for Collaborative European NeuroTrauma Effectiveness Research in Traumatic Brain Injury (CENTER-TBI) and 10.13039/501100008358Hersenstichting Nederland (Dutch Brain Foundation) for Neurotraumatology Quality Registry (Net-QuRe). Moreover, support by the 10.13039/501100008358Hersenstichting Nederland (Dutch Brain Foundation) was obtained with the Complement Inhibition: Attacking Overshooting inflammation @fter Traumatic Brain Injury (CIAO@TB) trial. Additionally, TvE received funding from the Niels Stensen Fellowship.

## Author contributions

Conceptualization, I.E, J.D. and W.C.; Methodology, I.E and J.D.; Validation, I.E, T.E, W.C. and J.D.; Formal Analysis, I.E.; Data Curation, J.D.; Writing – Original Draft Preparation, I.E. and J.D.; Writing – Review & Editing, T.E., E.K., M.J., W.M., W.C., J.D.; Supervision, W.C., J.D.; Funding Acquisition, I.E., T.E., W.C.

## Institutional review board statement

The study was conducted according to the guidelines of the Declaration of Helsinki, and approved by the local Ethics Committee of Leiden University Medical Center and Haaglanden Medical Center.

## Informed consent statement

Patient consent was waived due to the large percentage of patients that passed away and anonymization of data.

## Data availability statement

The database will be available upon reasonable request to the corresponding author. The statistical analysis plan and syntax will be made available upon request.

## Declaration of interests

The authors declare the following financial interests/personal relationships which may be considered as potential competing interests:

Wilco Peul reports a relationship with The 10.13039/501100000780European Union seventh Framework Program (grant 602,150) for Collaborative European NeuroTrauma Effectiveness Research in Traumatic Brain Injury (CENTER-TBI) that includes: funding grants. Wilco Peul reports a relationship with 10.13039/501100008358Hersenstichting Nederland (Dutch Brain Foundation) that includes: funding grants. Thomas van Essen reports a relationship with Niels Stensen Fellowship. that includes: funding grants.
